# Isolated Congenital Absence of Right Lower Lateral Cartilage and Reconstruction with Septal Cartilage: A Case Report and Review of Literature 

**DOI:** 10.29252/wjps.10.2.120

**Published:** 2021-05

**Authors:** Abolfazl Abbaszadeh, Ali Foroutan, Alireza Sherafat

**Affiliations:** 1 Fatima Plastic Surgery Hospital, Iran University of Medical Sciences, Tehran, Iran; 2 School of Medicine, University of Central Lancashire, Preston, United Kingdom

**Keywords:** Rhinoplasty, Absence of lower lateral cartilage, Cartilage reconstruction

## Abstract

Congenital anomalies of the nose are rare but may be associated with syndromes affecting craniofacial structures. Herein, we report a case of a congenital absence of lower lateral cartilage seen during an open rhinoplasty in a 23-yr-old lady with no underlying health conditions. Medical and surgical history were unremarkable and there were no evidences conducted of any previous traumatic facial injuries. During physical examination, a significant nostril asymmetry was noted to be present. In addition, cotton test showed no evidence of obstruction. The absence of lower lateral cartilage on the right side was noted during the degloving stage of the open rhinoplasty. Absence of lower lateral cartilage poses a technical challenge in surgery and in order to reconstruct this structure, cartilage can be harvested from concha, lower lateral cartilage, septum and cartilaginous dorsal hump during an open approach rhinoplasty.

## INTRODUCTION

Congenital nasal anomalies are often noted as part of a multisystemic syndrome affecting other organs as well as facial structures which can present as hypoplastic features^1^. Isolated presence of congenital nasal anomalies is very rare^2^. Their prevalence is estimated to be approximately 1 in 20,000 to 1 in 40,000 among those born alive^3^. In majority of cases, nasal cartilage defects are accompanied by other congenital craniofacial defects^4^.Teratogenic effects of some medications like Carbimazol may have a role in the etiology of nasal cartilage hypoplasia^5^. Previous studies have shown the use of harvesting cartilaginous tissue from lower lateral cartilage, conchal cartilage, septal cartilage, dorsal nasal hump cartilage or helical rim and conchal cartilage composite grafts^1^^,^^5^^-^^9^. 

Most of the times the defect is an incidental intra-operative finding when the patient is undergoing rhinoplasty either as an aesthetic surgery or due to a facial trauma. Herein, the patient was treated for cosmetic reasons. It is essential for surgeons to be aware of this condition and be prepared to reconstruct the lower lateral cartilage during the procedure.

## CASE PRESENTATION

The patient was a 23-yr-old woman, who came for cosmetic nose surgery without any complain of airway obstruction. She was treated at Fatima Plastic Surgery Hospital, Iran University of Medical Sciences, Tehran, Iran in 2020. 

The patient did not have any underlying health conditions and did not use any prescribed or over-the-counter medications. There was no history of any genetic conditions. The family history was also unremarkable. Moreover, there was no evidence of previous facial trauma, allergy or surgery as explicated by taking a comprehensive history. Psychiatric consultation was requested preoperatively in order to rule out any psychologic disorders such as body dysphoria which may affect the outcome of rhinoplasty. 

**Fig. 1:**
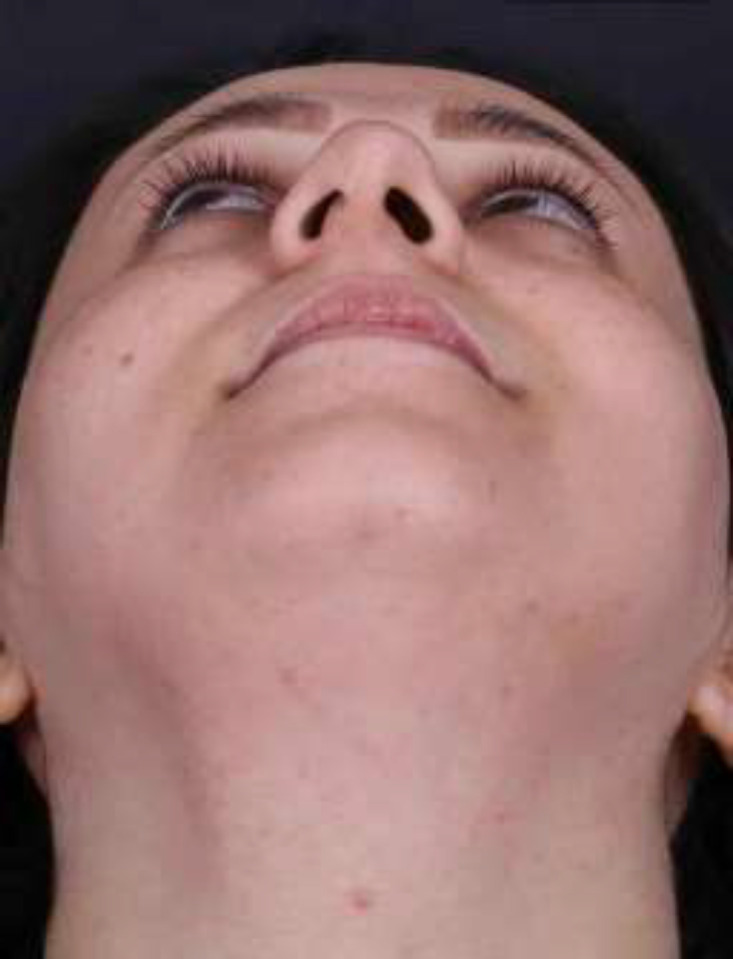
The pre-operative appearance of the asymmetrical nostrils

During the physical examination, cotton test showed no evidence of obstruction. There was a mild left septal deviation. An obvious nostril asymmetry was noticed (Figure 1). The history and physical examination were otherwise normal.

**Fig. 2:**
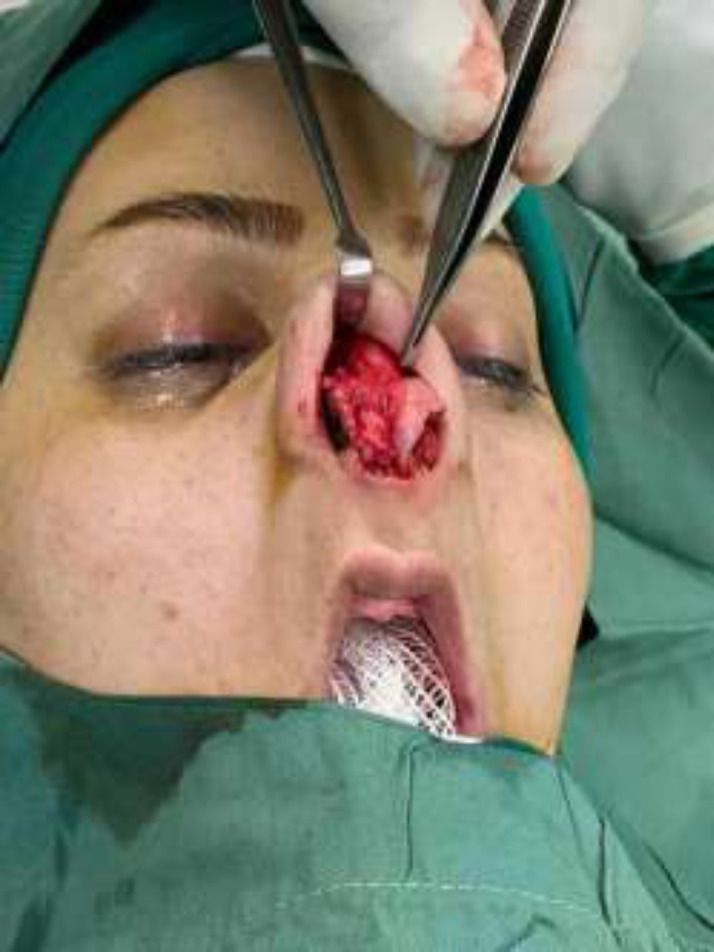
The intra-operative picture showing the absent Right Lower Lateral Cartilage

**Fig. 3:**
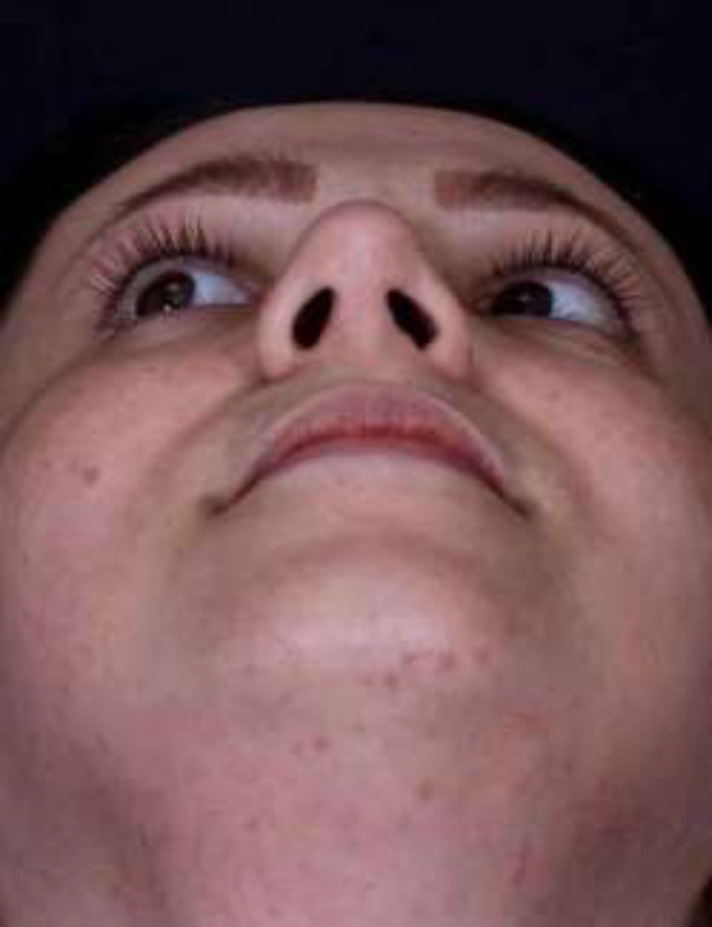
The post-operative outcome following septal reconstruction during an open approach rhinoplasty

Open rhinoplasty was planned for the patient. During the degloving, absence of lower lateral cartilage on the right side was discovered (Figure 2). Medial and lateral crura were reconstructed after harvesting septal cartilage, septoplasty was done, and tip graft was inserted to achieve an aesthetically acceptable outcome (Figure 3).

The patient was admitted to the ward for an overnight stay following the surgical reconstruction of the right lower lateral cartilage. She was discharged home the next day without any significant complications. Her recovery period was uneventful. Currently, she is under the specialist review every 3 months and no significant complications have been noted. She is not experiencing any symptoms during the day and at night. Finally, she is very satisfied with the current appearance of her nose and is in full compliance with the surgical team regarding her regular follow-up appointments. 

***Patient consent***

The patient provided a valid informed written consent for using her pictures and clinical notes in this case report. 

## DISCUSSION

The hypoplasia of nasal structure is frequently syndromic and rarely presents in isolation^10^. Nasomaxillary hypoplasia (binder syndrome), frontonasal dysplasia and medial cleft are among the syndromic causes^5^. 

 Absence of vomer or nasal bone has been reported in less than ten cases^11^^,^^12^. Columellar absences are usually syndromic^10^. Absence of Lower lateral cartilage is also extremely rare^2^. Since these anomalies would remain unknown to both the patient and the surgeon until confronting intraoperatively, they deserve special consideration. Surgeons should be aware of them prior to the surgery based on signs and be prepared to confront the absence of this structure^13^. 

Patients may seek surgery either due to functional airway obstruction or cosmetic complains.

Lower lateral cartilage has a mixed embryologic origin between 3^rd^ and 10^th^ week of gestation. The lateral crura develops from the lateral nasal process, but the medial crura stems from the medial nasal process. A vascular accident or a local pressure phenomenon could be the cause of this absence^2^.

Due to the surgeon’s discretion, septal cartilage was harvested to reconstruct the absent lower lateral cartilage on the right side in this case during the open approach rhinoplasty. However, as already mentioned in the introduction, there are various other sources of harvesting cartilage for reconstructing the defect shown by previous studies. Surgeons often have a preference of choosing a cartilage source based on their individual experience and the patients’ facial anatomy. Nevertheless, if the absence of the right lower lateral cartilage was detected prior to the surgery, the surgeon could get a chance to discuss their plan with the patient and involve her in the process of decision making on the basis of holistic patient-centered practice. Actively involving patients in the process of decision making for the choice of approach in cosmetic rhinoplasty is associated with a higher satisfaction rate among patients^14^. This would also be reassuring to the clinicians including the surgeon. 

The absence of lower lateral cartilage is rare, and the pathogenesis is not fully understood, however they can be easily addressed with open approach rhinoplasty. In majority of cases, those presenting with isolated absence of lower lateral cartilage have no symptoms pointing towards the early recognition of this defect. However, there are a few signs including having smaller nostrils, deeper alar crease and depressed dome that may prompt the assessment of patients for a possible absence of lower lateral cartilage prior to the surgery^13^. 

## CONCLUSION

Absence of lower lateral cartilage may be a rare incidental finding in non-syndromic patients without significant past medical history. Therefore, it is highly important to be aware of this rare condition and screen for that before the surgery. Noting the defect before the operation can guide surgeons in their choice of technique and reconstruction planning. Notably, the patients can be advised about risks and precautionary measures in advance of their surgery which would be important in terms of giving them the opportunity to provide a valid informed consent for open approach rhinoplasty and reconstruction. 
